# Dental Diplomas in the State of New York

**Published:** 1882-08

**Authors:** J. Edw. Line

**Affiliations:** Secretary of the Dental Society of the State of New York


### DENTAL DIPLOMAS IN THE STATE OF NEW YORK.

[Rochester, New York, July 10, 1882.]

EDITOR DENTAL REGISTER:—We find in the July number of the DENTAL REGISTER a communication on "Dental Diplomas in the State of New York," by "A Close Observer," who, if he observed anything at all, must have made his observations as through the bottom of a glass darkly; for it were simply impossible in a sober man to exhibit at one and the same time the amount and degree of ignorance displayed in this production, and the wanton perversion of truth that characterizes its every statement.

In answer to "How men of good common sense can look on and see the tendency of the working of such an affair as the New York Society has been carrying on in the diploma business, and not blush for the mischief being done," we submit that the implied charge that the society in question has ever been in the "diploma business," as "A Close Observer" would have that term understood, is false; that he knows it to be false by whomsoever made; that he knew it to be false at the time he put his words on paper; and we challenge him, or any one else possessing "moral courage" enough to make false statement, behind a *nom de plume*, to point out a single instance of "mischief" that New York's "M. D. S." has ever done. That it is "all wrong in principle," that there is "no need of such degrees," that they are "misleading," that they are "an insult to our schools of instruction," are mere opinions, and as such, coming as they do from this kind of "A Close Observer," deserve just a trifle more attention than if they had not found their way or been crowded into print.

The "sense of pity" he affects for those who have faced and passed the examination of our board of censors, "to see their good names so tarnished," is attenuated nonsense, and is shared only by other members of the clique of soreheads "A Close Observer" seems to so well represent. His prediction that "not many years will go by before some of these door-plates with D.D.S. (or M.D.) on them will be the passion of Peter

Funk of the junk shop" is important, and herein occurs to some for the first time; but its value as a prediction might have been greatly augmented had he added another, that of a number of idiots born into the world some years ago not a few died young, while others by some oversight on the part of a long-suffering providence, live to a good old age.

The statement that "My opportunities have not been very limited in my insight of the 'green-room' of diploma business" prompts him to suggest "moderation, but not fear, in attacking this matter," a suggestion he should have acted on years ago when he "was made a permanent member of the State Society," and when, as evidenced by his own confession in this very letter, instead of remaining to right what he believed to be a wrong, or even make the attempt to do so, he was so overwhelmed by that "moral courage" that he talks so much about and which is so characteristic of his ilk, that he sneaked out of the organization that through some ill-advised recommendation condescended, for the time being, to honor him with election to permanent membership, and has since, to all appearances, devoted his time and questionable talent to perfecting himself in the art of throwing mud.

He does not wish it understood that he has commented on this subject "for the sake of creating hot blood," but does it "hoping the knife of criticism will cut this blemish from an organization that has done, and can do, much that is helpful," which leads to the hope on our part that this knife may be larger and keener than that he uses, which seems to resemble a worn scalpel; while that, let him and his be assured, to be used in the return thrusts by the party attacked shall approach in size and execution the tool known as cleaver--shall be made ofthe material of fact sharpened by the desire and determination to get at and expose the true inwardness of the mixture of half-knowledge as to facts and culpability in the presentation of them that finds expression in such communications as this under consideration, and a fit mouthpiece in the persons of those who observe some things too closely.

In closing a rather extended notice of this slur, this libel, this attempt at defamation of one of the oldest, best organized and equipped of our State societies; this slanderous attack on our

Board of Censors; this effort to scandalize those who hold the "M.D.S.," let me say that the officers and members of the Dental Society of the State of New York are ever ready in their own and in the interests of those related to them, to resent and repel the unsupported and untrue statements of all persons whomsoever, known or nondescript, responsible or unreliable and and not to be trusted, when such statements appear in reputable journals. At the same time they will be found more ready, if possible, to discuss questions of society structure, of management or administration, with persons who are capable of distinguishing facts from their own imaginings; who have some notion what of truth is; and who have "moral courage" enough to declare and abide by that truth, at least when it will answer their purpose as well if not better than falsehood.

Finally, to forestall the objection on the part of some of our brother officers and fellow members, who are in nowise responsible for what we have said, that this notice of the work of "A Close Observer" hardly comports with the dignity looked for in an officer of so august a body, we answer that it is simply impossible to appear dignified while in the act of kicking a representative specimen of this particular variety of sorehead.

Very truly yours,

J. EDW. LINE, D.D.S., M.D.S.,
Secretary of the Dental Society of the State of New York.

